# Molecular Detection of *Acetobacter aceti* and *Acetobacter pasteurianus* at Different Stages of Wine Production

**DOI:** 10.3390/foods14010132

**Published:** 2025-01-05

**Authors:** Irina Mitina, Cristina Grajdieru, Rodica Sturza, Valentin Mitin, Silvia Rubtov, Anatol Balanuta, Emilia Behta, Angela Deaghileva, Fatih Inci, Nedim Hacıosmanoğlu, Dan Zgardan

**Affiliations:** 1 The Institute of Genetics, Physiology and Plant Protection, Moldova State University, 2002 Chisinau, Moldova; irina.mitina@sti.usm.md (I.M.); cristina.grajdieru@sti.usm.md (C.G.); valentin.mitin@sti.usm.md (V.M.); angela.deaghileva@sti.usm.md (A.D.); 2 Department of Oenology and Chemistry, Technical University of Moldova, 2004 Chisinau, Moldova; rodica.sturza@chim.utm.md (R.S.); silvia.rubtov@tpa.utm.md (S.R.); anatol.balanuta@enl.utm.md (A.B.); 3 Department of Preventive Medicine, State University of Medicine and Pharmacy of the Republic of Moldova, 2029 Chisinau, Moldova; emilia.timbalari@usmf.md; 4 National Nanotechnology Research Center (UNAM), Institute of Materials Science and Nanotechnology, Bilkent University, 06800 Cankaya, Ankara, Turkey; finci@bilkent.edu.tr (F.I.); n.haciosmanoglu@bilkent.edu.tr (N.H.)

**Keywords:** acetic acid bacteria, wine spoilage, primers, real-time PCR, volatile acidity

## Abstract

*Acetobacter aceti* and *Acetobacter pasteurianus* belong to acetic acid bacteria (AAB), associated with wine spoilage. The timely detection of AAB, thought essential for their control, is however challenging due to the difficulties of their isolation. Thus, it would be advantageous to detect them using molecular methods at all stages of winemaking and storage. In this paper, we analyzed wines, musts and grapes of 13 varieties grown in different regions with Protected Geographical Indication of the Republic of Moldova for the presence of AAB, *Acetobacter aceti* and *Acetobacter pasteurianus* by real-time PCR and measured wine volatile acidity. Overall, the AAB content in the mature wine explained 33.7% of the variance in the volatile acidity of the mature wine, while the *A. pasteurianus* content in the mature wine alone explained 59.6% of the variability in the volatile acidity in the wine, and its content in the grapes, must and wine explained about 70% of the variance in the the volatile acidity. This makes *A. pasteurianus* a good candidate to be a potential predictor of wine volatile acidity.

## 1. Introduction

*Acetobacter aceti* and *Acetobacter pasteurianus* belong to acetic acid bacteria (AAB)—a group of Gram-negative obligate aerobes that are associated with wine spoilage [1]. Their adverse impact on wine quality is attributed to their ability to convert carbohydrates and ethanol into acetic acid, which contributes to wine’s volatile acidity [2]. These *Acetobacter* species are found at all stages of winemaking, being a part of the natural microbial consortia on grape berry surfaces [3]. However, the dynamics of *A. aceti* and *A. pasteurianus* change compared to other AAB as the fermentation progresses. The initial stages of fermentation are characterized by a high diversity of AAB, with *A. aceti* and *A. pasteurianus* being underrepresented compared to *Gluconobacter oxydans*, the dominant AAB species in must. However, during the later stages of winemaking, the AAB diversity decreases, and *A. aceti* and *A. pasteurianus* dominate in mature wines [4,5,6]. This is explained by the fact that *Acetobacter* species are more tolerant to a higher ethanol content compared to *Gluconobacter* [7]. Acetic acid production is increased during must fermentation due to the competition between yeasts and bacteria in concurrent malolactic fermentation [8]. Small *A. aceti* and *A. pasteurianus* populations can survive during the fermentation stage, causing subsequent wine spoilage even after bottling [9]. Studies report that despite being an obligate aerobe, *A. pasteurianus* can survive for a prolonged time (about two months) under conditions of significant oxygen deficiency [10].

Volatile acidity is well known as a major indicator of wine spoilage. It consists principally of acetic acid with lower amounts of steam-distillable acids such as sorbic, formic, butyric and propanoic acid [2]. The European regulation (CE 1308/2013) [11] has set out limits for sale at 1.20 and 1.08 g/L of acetic acid for red wines and white/rose wines, respectively [11], as has the legislation of the Republic of Moldova [12]. These limits are provided by regulation regarding the organization of the wine market in the Republic of Moldova. Good cellar practices such as monitoring the sanitary state of grapes [13], high hygiene, microbiological control, oxygen limitation and reducing porous surfaces considerably reduce the risks of wine spoilage by acetic acid bacteria [14]. Because of the adverse impact of volatile acidity on wine quality and the role of *A. aceti* and *A. pasteurianus* in increasing wine volatile acidity, the monitoring and control of these species at every stage of winemaking is essential. However, assessing the levels of wine contamination with these bacteria using conventional platting and enrichment approaches are challenging [7]. Plate counting gives compromised results regarding cell counting, since AAB species are able to enter a viable but non-culturable state [15]. Bartowsky et al. mentioned the weak growth of AAB colonies isolated from wine, with them failing to propagate on different media [16]. Other authors [17] have used different enrichment media for AAB isolation (sterile must, sterile must with added ethanol and acetic acid, GYC medium, YPM medium and several others) and stated that not all of them supported bacterial growth. Colony formation and counting is also impeded by exopolysaccharide biofilms produced by some AAB species [17].

Molecular assays can overcome the limitations of conventional microbiological methods for AAB detection and quantification, since they are based on identifying a specific target sequence in the bacterial genome. Direct PCR permits identifying genomic regions of interest directly in the sample material, excluding the platting and enrichment step [18]. At the same time, molecular methods can be combined with traditional platting when the microbial concentration in the sample is low to reduce false-negative results (indirect methods) [19,20,21,22]. Despite having a lot of advantages, the PCR detection of spoilage microorganisms has certain drawbacks. The difficulties include cell lysis and nucleic acid extraction, cross-contamination and failed reactions due to the presence of inhibitory substances or competing DNA from non-target cells [23]. A major drawback is the inability of PCR to differentiate the DNA from dead and viable cells, and this is a critical factor for the food industry, regulatory agencies and consumers [24]. Apart from the drawbacks, PCR is still used as the gold standard for the detection of microorganisms, and it is utilized in this work as a major tool to monitor the presence of AAB during wine production. This could help prevent wine acetification caused by these species.

In this context, our study focuses on monitoring of the two major AAB species associated with wine spoilage, *A. aceti* and *A. pasteurianus*, at different stages of wine making. The main purpose is elucidating the individual contribution of each of the species to wine volatile acidity as a potential predictor of wine spoilage at the initial stages of fermentation.

## 2. Materials and Methods

### 2.1. Collection of Samples

Grape samples were collected from different regions with Protected Geographical Indication (PGI)—Codru, Stefan Voda and Valul lui Traian.

Three samples of each of the following grape varieties were used in this study at three stages of winemaking: Rkatsiteli, Feteasca Neagra, Augustina, Ametist, Feteasca Regala, Pinot Gris, Alexandrina, Nistreana, Viorica, Cabernet Petit, Rara Neagra, Feteasca Alba and Chardonnay (Table S1). They belong to four major groups: international varieties (Pinot Gris, Cabernet Petit, Chardonnay), local Georgian varieties grown in Moldova (Rkatsiteli), local Moldavian–Romanian varieties (Feteasca Neagra, Feteasca Alba, Feteasca Regala, Rara Neagra) and local Moldavian new selection varieties (Augustina, Ametist, Alexandrina, Nistreana, Viorica). The growth conditions of the grapes are outlined in Table S2.

The density of the musts at 20 °C was between 1.074 and 1.094 kg/m^3^, according to the standard OIV-MA-AS2-01B: R2009 [25]; the sugar content in the musts was 170–230 g/dm^3^, according to the standard OIV-MA-AS311-01A: R2009 [26]; the pH of the musts was in the range of 3.2–3.5, according to the standard OIV—MA-AS313-15: R2011 [27]; the titratable acidity of the wine was in the range of 5.4–8.0, recalculated in tartaric acid g/L according to the standard OIV—MA-AS313-01:R2015 [28]; the alcohol content in the wine was in the range of 10.00–13.5% vol.

Samples from the 2021 vintage were collected at three stages of winemaking: stage I—the collection and processing of grapes; stage II—must production, stage III—wine production after clarification and stabilization, before bottling.

Stage I—Grape surface. The vines were grown in the fields using standard practices without irrigation. Regular treatments against major fungal diseases were performed during the season. At the stage of maturity, the grapes had no visible symptoms of infection or damage. The grapes were collected manually at the stage of technical maturity and brought to the micro-winery facility of Technical University of Moldova. Immediately upon arrival, 150 g of grapes were washed in PBS buffer for 20 min, buffer was centrifuged at 5000× *g* for 20 min, pellet was collected and used for DNA extraction.

Stage II—Must. Grapes were destemmed and crushed using standard equipment in micro-winery of Technical University of Moldova. Selected EnartisFerm SC yeasts (Enartis©, Italy, Galliate) were added to the must or crushed grapes at a concentration of 20 g/hL. Must samples were taken for analysis before yeast inoculation.

Stage III—Wine. After alcohol fermentation, the white wines underwent sulfitation with potassium metabisulfite. Samples were taken for AAB detection three months after clarification and stabilization before bottling.

Regardless of the type of grape variety (international, local Moldavian–Romanian, local Moldavian new selection varieties, Georgian varieties), the main operations carried out to obtain the white wines were as follows [29]: harvesting grapes; destemming and crushing grape berries; maceration at t = 12–14 °C for 2–4 h, sulfitation 50–75 mg/dm^3^; must separation; clarification of the must for 18 h with bentonite administration at 0.5–1.0 g/dm^3^ at t = 10–14 °C; fermentation at t = 16 °C for 7–10 days; post-fermentation and wine formation at t = 10–12 °C; removal of the wine from the yeast lees with wine equalization and sulfitation at 10–20 mg/dm^3^; storage; integrated wine treatment (wine fining, racking, cold treatment at t = −3–5 °C for 5–7 days); cold filtration; wine resting for 10 days; membrane filtration; bottling and corking.

The operations carried out to obtain the red wines were as follows [29]: harvesting grapes; crushing and stemming grape berries; sulfitation 75 mg/dm^3^; maceration and fermentation at t = 26–28 °C for 4–6 days,; pressing grape mash; running off the free-run wine and the first fraction for additional fermentation; malolactic fermentation; wine formation on yeast lees; removal of wine from yeast lees with equalization and sulfitation (20 mg/dm^3^); storage; integrated wine treatment; wine fining for 10 days; racking; cold treatment at 3–5 °C for 5–7 days; cold filtration; wine resting for 10 days; membrane filtration; bottling.

### 2.2. Isolation of the Wine DNA

For DNA isolation from the grapes, a cell pellet obtained from washing 150 g of grapes was resuspended in 0.6 mL of extraction buffer, and further extraction was carried out following the same protocol as the must and wine samples [30].

For DNA isolation from the wine and must, 10 mL of each wine or must sample was centrifuged at 5000× *g* for 30 min, followed by pellet resuspension in 0.6 mL of the extraction buffer (Tris–HCl 0.2M pH 8.0, NaCl 0.25M, Na_2_EDTA 0.025M, SDS 5% *w*/*v*). Then, the cell suspension was heated at 65 °C for 1 h. All reagents were of a molecular biology grade (Sigma-Aldrich, Burlington, MA, USA). Then, 60 mg of PVP powder and a 0.5 volume of ammonium acetate solution (7.5 M) were added to the sample and incubated on ice for 30 min, followed by a 10 min centrifugation at 10,000× *g*. Then, the supernatant was transferred to a fresh tube, mixed with an equal volume of chloroform, vortexed and centrifuged again at 10,000× *g*. The upper phase was carefully collected and transferred to a new tube, mixed with an equal volume of isopropanol and incubated at −20 °C for 30 min. The samples were centrifuged, and the pellet washed twice with 70% ethanol and air-dried, and the purified DNA was dissolved in 50 μL of water; then, 2 μL of the resulting DNA solution was used for each PCR reaction. DNA quality and concentration were checked spectrophotometrically using a Genova Nano micro-volume spectrophotometer. Three aliquots of each sample were taken for DNA extraction.

### 2.3. Real-Time PCR Amplification

Polymerase chain reactions (PCRs) were conducted via a CFX96 TouchTM BIORAD real-time PCR detection system. The PCR cycling conditions were as follows: 95 °C for 2 min as an initial denaturation step followed by alternations of 95 °C for 15 s and 60 °C for 1 min for 35 cycles in case of *A. aceti* and *A. pasteurianus* detection, and alternations of 95 °C for 15 s and 65 °C for 1 min for 35 cycles in case of AAB screening. For melting curve construction, samples were heated to 95 °C for 15 s, then incubated at 60 °C for 1 min (1.6 °C/s ramp rate), and then heated to 95 °C for 15 s (0.15 °C/s ramp rate). The detection of the amplified product was performed via the SYBR channel.

For screening for the presence of acetic acid bacteria (total AAB), previously published primers targeting the 16S rRNA region capable of the detection of multiple AAB, including six *Acetobacter* and seven *Gluconobacter* species, were used. The primer characteristics were as follows: K-AAB_F: 5′-AAGGGGGCTAGCGTTG CTCG-3′ (20 bp; forward primer; melting temperature = 64.98 °C; GC contents = 65%) and K-AAB_R: 5′-ACCGCCTACACGCCCTTTACG-3′ (21 bp; reverse primer; melting temperature = 65.01 °C; GC contents = 61.9%). The amplicon size was 52 bases [31].

Previously described primers based on the sequence AB161358.1 (*A. aceti* genes for 16S rRNA, 16S-23S rRNA ITS and 23S rRNA) were used for *A. aceti* detection [30]. The primer characteristics were as follows: P173—5′-TTTTGAAATGTGACGCGCTTGAATG-3′ (25 bp; forward primer; melting temperature = 62.01 °C; GC content = 40%) and P174—5′-TTGCTCCCATGCACAGAAACC-3′ (21 bp; reverse primer; melting temperature = 61.43 °C; GC content = 52.38%). The amplicon size was 96 bases [30].

Previously described primers based on the sequence AJ888874.1 (*A. pasteurianus* partial adhA gene for alcohol dehydrogenase) were used for *A. pasteurianus* detection [24]. The primer characteristics were as follows: P175—5′-CCGGCGGTGATCTTCTGTTC-3′ (20 bp; forward primer; melting temperature = 61.08 °C; CG content = 60%) and P176—CCGCTCTGTGCGTCAAACTT-3′ (20 bp; reverse primer; melting temperature = 61.50 °C; CG content = 55%). The amplicon size was 100 bases [30].

### 2.4. Calculations of Delta Cq Values

qPCR cycle threshold (Cq) values represent the number of amplification cycles required for the fluorescent signal to exceed the basal threshold level. Cq values are inversely related to the number of copies of the target gene in a sample, meaning that lower Cq values correlate with higher pathogen loads [32]. Nevertheless, since these values are inversely correlated with the amount of the target organism, they can be difficult to interpret. In addition, it is not always necessary to know the exact amount of the target organism for the particular experimental purpose; rather, a comparative study of the infection load between samples may be quite informative. To simplify and visualize the data regarding the infection load in different samples, we analyzed the qPCR data by calculating the delta Cq value by subtracting the Cq value obtained for a given sample from Cq value = 35, which is the number of cycles in the PCR reaction and corresponds to the minimal amount of target gene that can be detected in this assay by the following formula:

delta Cq = Cq_max_ − Cq_sample_,

where Cq_max_ is the number of PCR cycles, and Cq_sample_ is the actual Cq number of the sample.

Thus, delta Cq is the difference between the actual Cq value and the Cq value of 35, and it indicates how sooner the fluorescent signal exceeds the threshold level in the sample compared to the theoretical minimal amount corresponding to 35 cycles. The greater the difference, the more of the target gene there initially was contained in the sample and the higher the infection load was in the sample.

Negative samples were considered to have delta Cq = 0.

### 2.5. Measurement of the Volatile Acidity in Wine

Volatile acidity was determined via steam distillation/titration using the method OIV-MAAS313-02: R 2015 from the Compendium of International Methods of Analysis—OIV [33].

### 2.6. Statistical Analysis

The experiments in this study were performed in triplicate. One-way analysis of variance (ANOVA) was performed followed by Tukey’s test at a significance level of *p* ≤ 0.05 or *p* ≤ 0.01 using the Minitab 17 Statistical software (Minitab, LLC, 2021. Minitab, Available at: https://www.minitab.com). Simple and multiple linear regression tests were performed at a significance level of *p* ≤ 0.05 to assess the relationship between *Acetobacter* quantity in the must and wine samples and the levels of volatile acidity in the respective wine samples.

## 3. Results and Discussion

### 3.1. Distribution of AAB, A. aceti and A. pasteurianus in Wine Samples at Different Stages of Winemaking

In this work, we studied the distribution of total AAB and two *Acetobacter* species (*A. aceti* and *A. pasteurianus*) in wine samples at different stages of wine making: grape berries, must and wine. The primers for AAB detection were designed to a conserved segment encoding *Acetobacter* 16S rRNA, giving a positive signal for *Acetobacter aceti*, *Acetobacter estunensis*, *Acetobacter liquefaciens*, *Acetobacter orleanensis*, *Acetobacter pasteurianus*, *Acetobacter persicus*, *Gluconacetobacter hansenii*, *Gluconacetobacter medellinensis*, *Gluconacetobacter xylinus*, *Gluconobacter cerinus*, *Gluconobacter frateurii*, *Gluconobacter morbifer* and *Gluconobacter oxydans*. For all of these species, the melting temperature of the amplified fragment was the same, 81.5 °C [31]. Thus, PCR amplification with this primer pair can detect the presence of one or several of AAB; however, it cannot discriminate between species and cannot identify which species and how many of them are present. Thus, even though these primers cannot assess the biodiversity of the AAB present in the samples, they are a very valuable tool for detecting the presence of AAB and assessing the level of AAB contamination.

Negative control samples (sterile wine) did not give a Cq value during 35 cycles of amplification.

**Figure 1:**
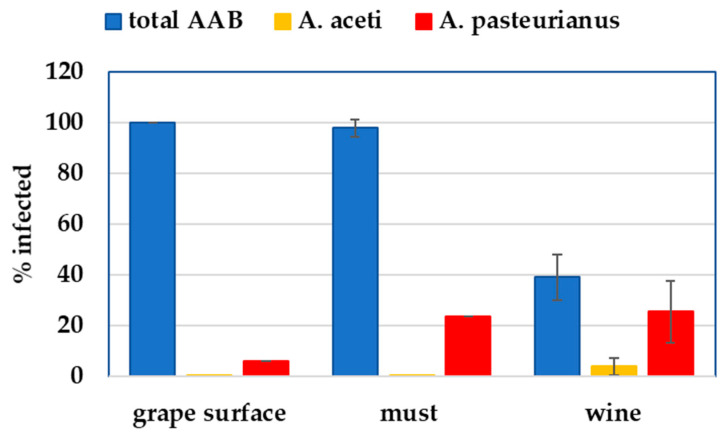
Frequency of AAB, *A. aceti* and *A. pasteurianus* in grape, must and wine. Dark blue color: total AAB detected; ochre color: *A. aceti* detected; red color: *A. pasteurianus* detected.

Interestingly, 100% of the grape surface wash, 94.1% of the must and 39.2% of the wines were positive for total AAB; 5.8% of the grapes, 23.5% of the must and 25.5% of the wine samples were infected with *A. pasteurianus*, while none of the grape or must samples were infected with *A. aceti*, and only 3.9% of the wines were infected with this wine spoiler at the levels detectable at 35 PCR cycles. This was unexpected, since *A. aceti* is a known wine spoiler that is responsible for wine volatile acidity [34]. This can be explained by the effect of the year, since grapes from a one-year vintage were studied, or by some interspecific competition between the AAB species. Figure 1 depicts the distribution of the total AAB species, *A. pasteurianus* and *A. aceti* in the grape, must and wine samples, as well as the frequencies of the samples testing positive for the given wine spoiler at each stage of winemaking.

Due to the small number of samples infected with *A. aceti*, as well as its apparently minor effect on wine volatile acidity, the following discussion focuses on total AAB and *A. pasteurianus*.

**Figure 2:**
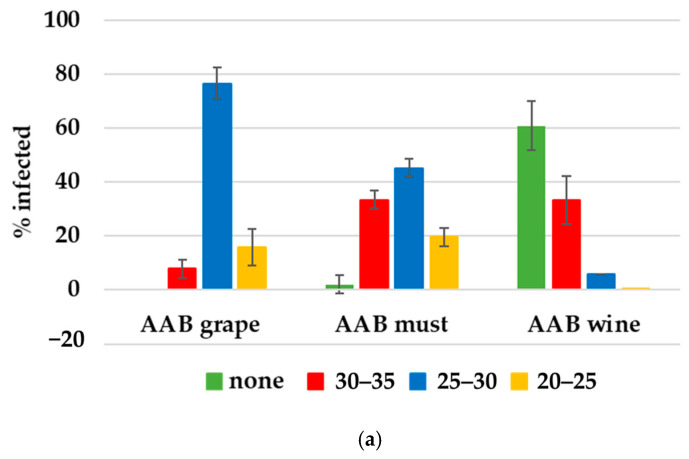

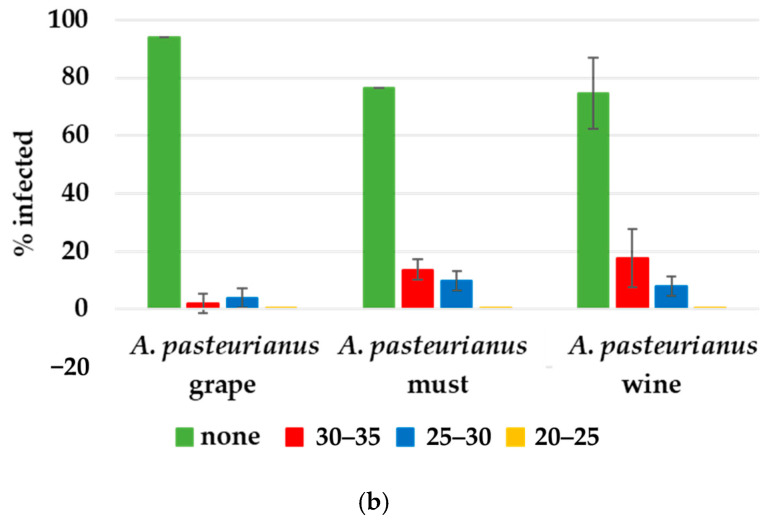
Distribution of infection load of total AAB species (**a**) and *A. pasteurianus* (**b**) in grape, must and wine samples. Green color: none detected; red color: low infection load, Cq values 30–35; blue color: medium infection load, Cq values 25–30; ochre color: high infection load, Cq values 20–25.

**Figure 3:**
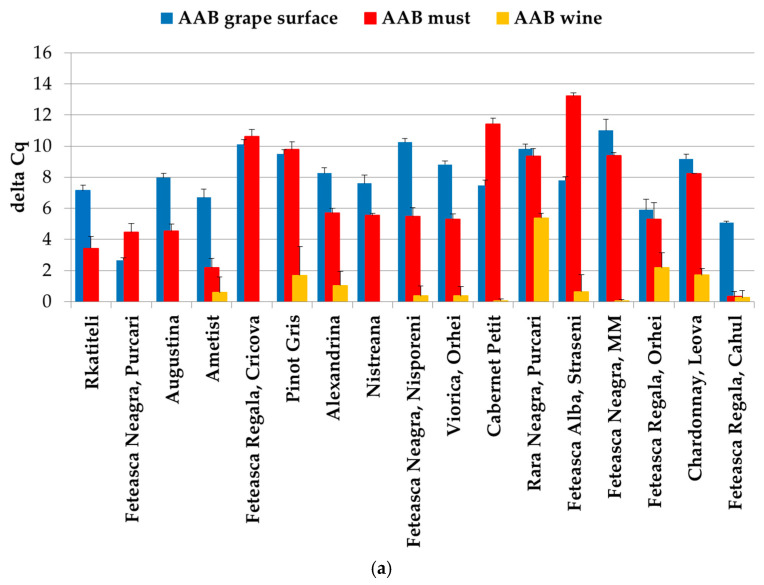

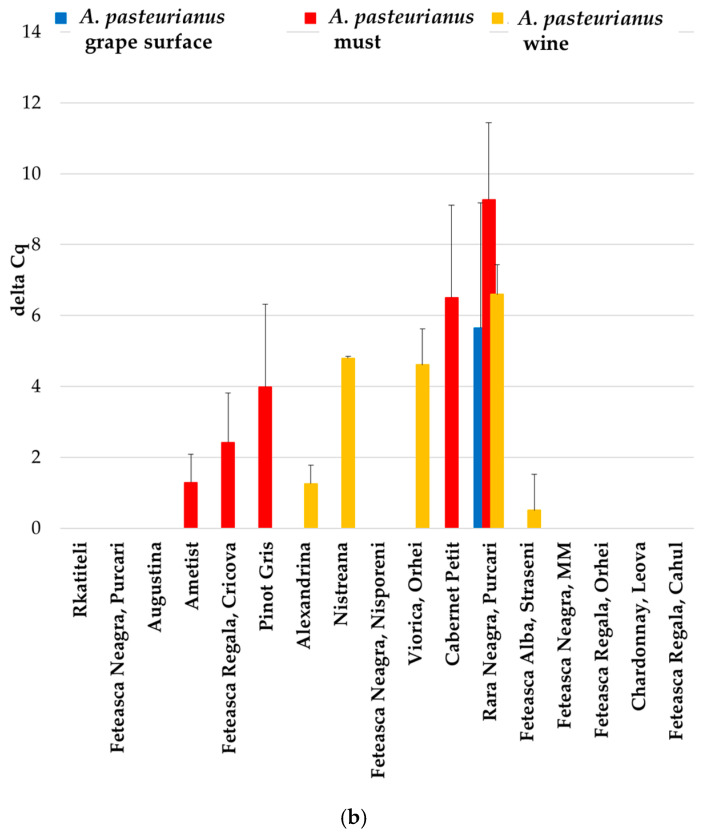
Distribution of infection load of total AAB species (**a**) and *A. pasteurianus* (**b**) in individual samples of grape, must and wine. Dark blue color: grape surface; red color: must; ochre color: wine.

Various data suggest a correlation between the Cq value and the infection load of the samples [32,35]. Figure 2 demonstrates the infection load of the grape, wine and must samples with total AAB and *A. pasteurianus*. Curiously, the samples of the grape surfaces not only had a 100% frequency of infection with AAB, but they also had a high infection load (92.1% of all samples from grape surfaces had a Cq value < 30). Overall, the infection load with AAB dropped in the must samples, where 64% of the samples had a Cq value < 30, and it further decreased in the wine samples, where only 5.8% of the samples had an infection load corresponding to a Cq value < 30 (Figure 2a). The overall delta Cq values of AAB in the grape surface samples were significantly higher than that in the must (*p* < 0.05) and wine (*p* < 0.01) samples, indicating a significantly higher infection load of acetic acid bacteria in the earlier stages of winemaking. Similarly, the overall delta Cq values of AAB in the must samples were significantly higher (*p* < 0.01) than in the wine samples, fitting the above observation. This tendency can be further seen in Figure 3a, depicting the distribution of AAB in individual samples at different stages of winemaking. Both the number of samples infected with total AAB and the delta Cq values of those infected samples decreased dramatically in the wine compared to in the must and grape surface samples. This is consistent with the previously described fact that the acetic acid bacteria population is highly reduced during must fermentation.

However, in this study, this did not seem to be the case for *A. pasteurianus*. In this case, though both the frequency of infected samples and the infection load increased from 4% infected samples with a Cq value < 30 in the grape surface wash to 9.8% infected samples with a Cq value < 30 in the must and 7.8% with a Cq value < 30 in the wine (Figure 3b). However, this change was not significant at *p* < 0.05. These data imply that in the earlier stages of winemaking, AAB species other than *A. aceti* and *A. pasteurianus* dominated; however, their number dropped below the detectable level in the later stages, and they did not seem to have a major effect on the wine volatile acidity at stage III, i.e., wine. Even though the acetic acid bacteria typically associated with grapes and must are known to be *Gluconobacter oxydans* [3,9,12], we could stably detect *A. pasteurianus* in 5 out of the 17 analyzed must samples. Moreover, two of these samples had a Cq value < 30, corresponding to a rather high content of these bacteria. A possible explanation is the early sampling of the must, before active fermentation started.

In addition, *A.pasteurianus* appeared in the wine samples, even though it had not been detected in the corresponding must samples. This was the case of Alexandrina, Viorica, Feteasca Alba and Nistreana (Figure 3b). Interestingly, all of these are white wines. A possible explanation would be that these musts were infected with a low amount of *A. pasteurianus*, below the detection levels, and once fermentation was completed and the environment became favorable, their active growth started. Alternatively, the infection could have occurred at the winemaking site, or their active growth could have been boosted by some winemaking practices [1]. For example, equipment (barrels, pumps, presses, filters, etc.) could have been contaminated with these bacteria (REF), or the wines could have been cross-contaminated by air circulation [36] or a vector, e.g., a fruit fly [37]. Another possibility is the presence in low amounts of some strains capable of surviving in unfavorable fermentation conditions, who started to actively grow after fermentation ended.

Considering the stage of winemaking at which the infection with *A. pasteurianus* occurred, only one variety (Rara Neagra: 5.8%) had detectable amounts of this wine spoiler at all analyzed stages, i.e., I, II and III (I—grape surface, II—must, III—wine) (Figure 3b).

In four samples (Alexandrina, Nistreana, Feteasca Alba-Straseni and Viorica Orhei) infection with *A. pasteurianus* occurred only at the wine stage; in four samples (Ametist, Feteasca Regala-Cricova, Pinot Gris, Cabernet Petit), only the musts had detectable levels of infection. This may indicate that a detectable amount of *A. pasteurianus* was introduced into the winemaking process on the grape surface only in the case of Rara Neagra; in the rest of the cases, the infection could be detected at the stage of must or wine.

**Figure 4:**
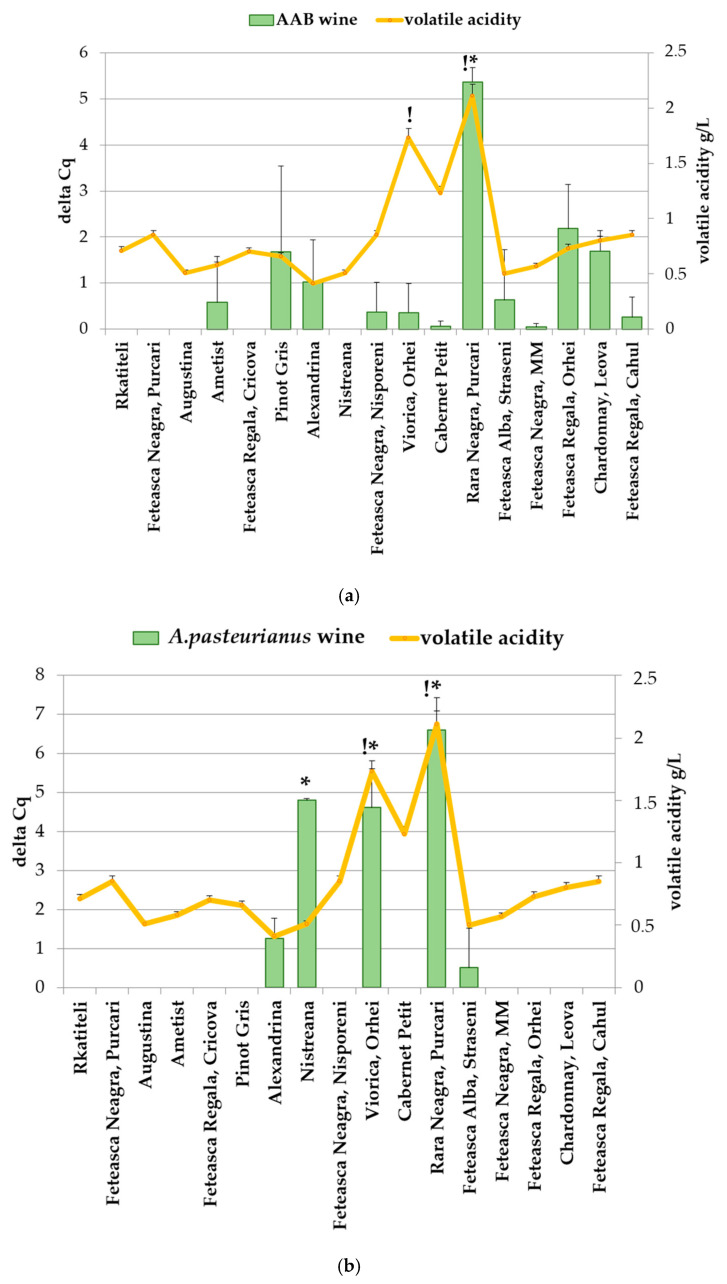
Distribution of total AAB (**a**) and *A. pasteurianus* (**b**) in wine and volatile acidity of wine samples. One-way analysis of variance (ANOVA) followed by Tukey’s test was performed. Asterisk (*) indicates statistical significance (*p* < 0.05). Volatile acidity of wine samples expressed as acetic acid. The admissible limits are 1.08 g/L for white wines and 1.2 g/L for red wines. *p* ≤ 0.05. Exclamation mark (!) indicates the wine samples exceeding admissible limits for corresponding volatile acidity.

Figure 4 shows the distribution of total AAB (Figure 4a) and *A. pasteurianus* (Figure 4b) in the wine and must samples, expressed as the delta Cq values of the samples, as well as the volatile acidity of the wine samples.

The Rara Neagra wine was affected at all three stages (grape surface, must and wine), and it had the highest delta Cq value in the must and wine. Relatively low Cq values were observed in Viorica (wine), Cabernet Petit (must) and Feteasca Neagra-MM (must).

Three wine samples (Viorica, Nistreana and Rara Neagra) contained the most *A. pasteurianus* DNA out of all the samples. Since *A. pasteurianus* produces acetic acid and acetic acid is the main constituent of wine volatile acidity [9], the volatile acidity of the wine samples was measured.

Most of the wine samples had a volatile acidity within the admissible limits. The volatile acidity of two wine samples (Rara Neagra and Viorica) exceeded the admissible limit. Interestingly, the same two wine samples (Rara Neagra and Viorica) had the highest content of *A. pasteurianus*. Comparing Figure 3b and Figure 4b, it is noticeable that the wine with the highest volatile acidity, namely Rara Neagra, had the highest delta Cq value for *A. pasteurianus* in both the wine (delta Cq = 6.6 ± 0.83) and must (delta Cq = 9.35 ± 0.49) samples as well as the highest delta Cq value (deltaCq = 5.37 ± 0.31) for total AAB in the wine (Figure 3a), indicating the highest infection load of all the samples, and it was also the only sample where *Acetobacter* was detected on the grape surface. Another wine exceeding the admissible limit for volatile acidity, namely, Viorica, had a delta Cq = 4.61 ± 1.01 in the wine for *A. pasteurianus* (Figure 3b and Figure 4b). However, this wine did not have a high Cq value for total AAB in either the wine (delta Cq = 0.36 ± 0.59) (Figure 3b and Figure 4b) or the must (delta Cq = 5.29 ± 0.35) (Figure 3b). One sample (Cabernet Petit) had a marginal volatile acidity at the admissible limit. This sample had a relatively high delta Cq value for both *A. pasteurianus* (delta Cq = 6.51 ± 2.59) and total AAB (delta Cq = 11.42 ± 0.38) in the must, but these microorganisms were not detected in the wine, possibly due to the wine treatment or competition with other wine microorganisms.

These data suggest that *A. pasteurianus* could be at least partially responsible for increasing the volatile acidity of these wines above the admissible limits. The same conclusion was reached by the authors of [16], who established that a closely related group of *A. pasteurianus* predominated in isolates from wines with an increased volatile acidity, as detected via an analysis of the 16S rRNA region and RAPD-PCR. Thus, *A. pasteurianus* can be considered the species responsible for wine alteration [16]. This may have potential implications in the search of microorganisms for bioprotection against acetic acid bacteria [38].

Another notable thing is the divergence in the results of the content of acetic acid bacteria in the wine, as detected by different primer pairs. Thus, of all the wine samples, only Rara Neagra showed a high level of both *A. pasteurianus* (delta Cq = 6.6 ± 0.83) and total AAB (delta Cq = 5.37 ± 0.31), while Alexandrina and Feteasca Alba-Straseni showed a low infection rate with both *A. pasteurianus* (delta Cq = 0.83 ± 0.81 and delta Cq = 0.41 ± 0.71) and total AAB (delta Cq = 1.03.83 ± 0.90 delta Cq = 1.89 ± 0.63), respectively (Figure 4a,b). The moderate levels of AAB in the wines of Feteasca Regala Orhei, Chardonnay and Pinot Gris and the absence of detectable levels of *A. pasteurianus* and *A. aceti* in these samples could indicate that these wines have some other species of acetic acid bacteria, detectable by the primers for AAB screening. The high infection levels in the Viorica wine sample of *A. pasteurianus* (delta Cq = 4.61 ± 1.00) and the relatively low infection level of total AAB (delta Cq = 0.36 ± 0.62) could indicate that most of the AAB present in this wine was *A. pasteurianus*. The higher levels of *A. pasteurianus* (delta Cq = 5.54 ± 0.12) in the Nistreana sample and the lack of any detection of total AAB is of a greater concern. One potential explanation is that the primer pair specific for *A. pasteurianus* detection recognized the bacteria present in this wine sample but it was not recognized by the pair of primers for AAB screening. This could either be because the AAB screening pair did not recognize the particular *A. pasteurianus* strain present in Nistreana wine or because the primer pair for *A. pasteurianus* recognized a bacteria with a high degree of similarity to the *A. pasteurianus* sequence.

### 3.2. Dependance of Wine Volatile Acidity on the Presence of Acetic Acid Bacteria in the Samples

The dependence of the volatile acidity in wine on the quantity of acetic acid bacteria (*A. pasteurianus* and total AAB) in the must or wine samples (expressed as delta Cq) was investigated by simple and multiple regression.

The results of the multiple linear regression indicated that there was a moderate collective significant effect of total AAB propagation in the grapes, must, and wine on the volatile acidity (F(1, 15) = 7.63, *p* = 0.015, R^2^ = 0.34, R^2^_adj_ = 0.29). Overall, the AAB (total AAB) content in the wine explained 33.7% of the variance in the volatile acidity of the mature wine (R^2^ = 0.337). The total AAB contents, expressed as the delta Cq value, in the grapes and must were not significant predictors for volatile acidity.

On the contrary, the impact of *A. pasteurianus* on the wine volatile acidity was very strong—overall, the *A. pasteurianus* content in the grapes, must, and wine, expressed as the delta Cq value, explained about 70% of the variance in the volatile acidity (F (2,14) = 17.86, *p* < 0.001, R^2^ = 0.72, R^2^_adj_ = 0.68). The *A. pasteurianus* content in the mature wine alone explained 59.6% of the variability in the volatile acidity in the wine (R^2^ = 0.595). The content of *A. pasteurianus* in the grapes was not a significant predictor of volatile acidity. Therefore, monitoring of *A. pasteurianus* in must and wine during winemaking might give significant data for forecasting the volatile acidity of mature wine.

A major limitation of the study is its relatively small number of varieties (*n* = 17) and a single year of observations. It would be interesting to broaden the spectrum of the analyzed varieties and detected AAB species. This would shed light on the role of possible interspecific competition between various AAB species at different stages of winemaking in wine volatile acidity. In addition, it would be good to continue observations during various years. More observations are needed to explain the low levels of *A. aceti* and its rare presence in the samples, especially the wine samples.

## 4. Conclusions

Overall, this research showed a tendency of decreasing the content of total AAB during the later stages of wine making.

The dynamics of *A. pasteurianus* showed a higher infection level in musts and wines, and more samples were infected with these bacteria.

Contrary to expectations, in this study, *A.aceti* did not have a major effect on wine volatile acidity.

Monitoring of AAB, especially *A. pasteurianus*, at different stages of wine production could help in forecasting the increase in the volatile acidity of mature wines.

Apparently, the presence of solely *A. pasteurianus* in must and wine contributes significantly to wine volatile acidity. This species could be a potential predictor of increases in wine volatile acidity.

Identifying the major AAB species responsible for the increase in volatile acidity could help in the search for microorganisms for bioprotection against wine acetification.

## Data Availability

The original contributions presented in this study are included in the article and Supplementary Materials. Further inquiries can be directed to the corresponding author.

## Supplementary Materials

The following supporting information can be downloaded at: https://www.mdpi.com/article/10.3390/foods14010132/s1, Table S1. Samples analyzed in this study. Table S2. Vine growing conditions in different regions in 2021.
