# Supplementary material for: Molecular Detection of Acetobacter aceti and Acetobacter pasteurianus at Different Stages of Wine Production
Source: Foods. 2025 Jan 5;14(1):132. doi: 10.3390/foods14010132 (PMC11720281; doi:10.3390/foods14010132)
Supplement: Supplementary file 1 [file foods-14-00132-s001.zip › foods-3383419-supplementary.pdf]

Table S1. Samples analyzed in this study.

Nr.	Varieties	PGI	Type of variety
1	Rkatsiteli	Codru	Georgian varieties
2	Feteasca Neagra, Purcari	Stefan Voda	Local Moldavian–Romanian varieties
3	Augustina	Codru	Local Moldavian new selection varieties
4	Ametist	Codru	Local Moldavian new selection varieties
5	Feteasca Regala, Cricova	Codru	Local Moldavian–Romanian varieties
6	Pinot Gris	Codru	International varieties
7	Alexandrina	Codru	Local Moldavian new selection varieties
8	Nistreana	Codru	Local Moldavian new selection varieties
9	Feteasca Neagra, Nisporeni	Codru	Local Moldavian–Romanian varieties
10	Viorica	Codru	Local Moldavian new selection varieties
11	Cabernet Petit	Codru	International varieties
12	Rara Neagra	Stefan Voda	Local Moldavian–Romanian varieties
13	Feteasca Alba	Codru	Local Moldavian–Romanian varieties
14	Feteasca Neagra, Milestii Mici	Codru	Local Moldavian–Romanian varieties
15	Feteasca Regala, Orhei	Codru	Local Moldavian–Romanian varieties
16	Chardonnay	Valul lui Traian	International varieties
17	Feteasca Regala, Cahul	Valul lui Traian	Local Moldavian–Romanian varieties

Table S2. Vine growing conditions in different regions in 2021.

Nr.	Varieties	PGI	Soil	Altitude	Mean air, °C	Annual precipitation, mm
1	Rkatsiteli	Codru	Typical chernozem, weakly humiferous and carbonate	122	10.6	490
2	Feteasca Neagra, Purcari	Stefan Voda	Carbonate chernozems, weakly eroded, loamy-clayey	103	11.4	490
3	Augustina	Codru	Ordinary, loamy chernozems	126	10.6	666
4	Ametist	Codru	Ordinary, loamy chernozems	126	10.6	666
5	Feteasca Regala, Cricova	Codru	Typical chernozem, weakly humiferous and carbonate	122	10.6	666
6	Pinot Gris	Codru	Leached, loamy-clayey chernozems	28	10.6	666
7	Alexandrina	Codru	Ordinary, loamy chernozems	126	10.6	666
8	Nistreana	Codru	Ordinary, loamy chernozems	126	10.6	666
9	Feteasca Neagra, Nisporeni	Codru	Carbonate chernozems, moderately eroded, clayey-loamy	91	10.6	666
10	Viorica	Codru	Typical gray soils	68	10.6	666
11	Cabernet Petit	Codru	Leached, loamy-clayey chernozems	28	10.6	666
12	Rara Neagra	Stefan Voda	Carbonate chernozems, weakly eroded, loamy-clayey	103	11.4	490
13	Feteasca Alba	Codru	Ordinary, loamy chernozems	90	10.6	666
14	Feteasca Neagra, Milestii Mici	Codru	Carbonate chernozems, weakly eroded, loamy-clayey	109	10.6	666
15	Feteasca Regala, Orhei	Codru	Typical gray soils	68	10.6	666
16	Chardonnay	Valul lui Traian	Carbonate, clayey chernozems	39	11.4	490
17	Feteasca Regala, Cahul	Valul lui Traian	Carbonate, clayey chernozems	52	11.4	490
